# Changes in bubble-cloud dissolution throughout the application of histotripsy pulses

**DOI:** 10.1103/9vvg-yfmb

**Published:** 2025-10-16

**Authors:** Michael Gomez, Katia Flores Basterrechea, Muskan Singh, Himanshu Shekhar, Kenneth B. Bader

**Affiliations:** 1 Department of Physics, University of Chicago, Chicago, Illinois 60637, USA; 2 Department of Radiology, University of Chicago, Chicago, Illinois 60637, USA; 3 Department of Electrical Engineering, Indian Institute of Technology Gandhinagar, Gandhinagar, India

## Abstract

Material damage is a consideration for any applications where pressure fields may initiate cavitation. Histotripsy is a focused ultrasound therapy that exploits the destructive property of bubble clouds to disrupt viscoelastic tissue. The resulting effect achieves the same outcomes as surgery without the need for an invasive procedure. This study investigated the relationship between changes in bubble clouds throughout focused ultrasound exposure. Specifically, the dissolution period of bubbles generated in water and agarose gel was tracked with ultrafast ultrasound imaging. This imaging marker was found to increase in agarose during the initial stages of exposure, and maintained steady-state values after 555 ± 64 applied histotripsy pulses. The relative change in damage area of the gel and bubble-dissolution period with exposure duration were found to be equivalent (*p* > 0.05). Acoustic emissions tracked with passive acoustic imaging were also evaluated and were found to have a different functional form with exposure duration than the bubble-dissolution period or area of damage to the gel. In contrast, there were no noted changes in these imaging markers throughout histotripsy exposure when bubble clouds were generated in water. A previously validated bubble-dynamics model was extended to calculate the anticipated changes to bubble expansion during histotripsy exposure under the assumption that the agarose elastic modulus approached 0 kPa (i.e., total fractionation). The calculations predicted larger changes in the bubble dynamics than was observed, which might reflect complicated dynamics of the bubble cloud during the final stages of treatment.

## I. INTRODUCTION

Cavitation refers to the formation, growth, oscillation, and/or collapse of bubbles. These actions occur when a sufficient negative pressure is applied to compressible impurities in an otherwise homogenous fluid or viscoelastic medium [1]. Multiple fields are impacted by micro-and millimeter-sized cavitation bubbles, including manufacturing, oceanic noise, and ultrasound [2]. Investigations into bubbles were initiated by John William Strutt (Lord Rayleigh) to address the erosion of ship propellers [3]. He identified that the energy density initiated during cavitation was sufficient to damage even the hardest materials [4]. Subsequent studies have confirmed the findings of Strutt though observations of bubble-induced shock waves and light emissions [5,6].

The deformation of soft materials by cavitation is important for many applications, including rheology, chemical processing, and botany [7,8]. Biomedical ultrasound devices have been developed that rely on bubbles for therapeutic benefit. Histotripsy is a focused ultrasound therapy for tissue ablation [9]. The outcome of a histotripsy procedure is equivalent to surgery without the need for an incision [10]. The technology was cleared by the U.S. Food and Drug Administration in October 2023 for the routine treatment of liver tumors. Additional trials are also underway to apply histotripsy to the kidneys and pancreas [11].

The ultrasound pulse itself is not directly responsible for damage. Rather, the pressure field initiates a specific type of cavitation in which a cloud of bubbles strain tissue to the point of fractionation [12]. Bubbles within the cloud expand throughout the ultrasound pulse [13], after which they undergo a strong collapse and rebound [14]. Bubble clouds have been observed to persist for hundreds of milliseconds after formation [15]. The gaseous and vaporous bubble contents increase rapidly during application of the ultrasound pulse and may contribute to the observed persistence of the cloud [16].

Imaging methods are needed to adjust the histotripsy exposure in real time as required to ensure successful outcomes. Approaches that provide direct visualization of the tissue can be ambiguous or are applied after the procedure has already concluded [17]. A potential alternative approach is to monitor histotripsy to assess changes in the bubble cloud over the course of treatment. Indeed, prior investigations have demonstrated that tissues exposed to histotripsy undergo softening due to repeated exposure [18,19], consistent with an increase in the local diffusivity [20]. These outcomes coincide with modulation of the observed maximum bubble size, shock amplitude, and lifespan, as detected through high-speed optical imaging and acoustic emissions [21].

Ultrafast ultrasound imaging provides additional information about changes in the bubble-cloud scattering intensity with submillisecond temporal resolution [15]. This timeframe is sufficient to quantify the dissolution period of the cloud [22], which is linked to bubble size [23]. Prior investigations have assessed the relative change in bubble-cloud dissolution based on pulsing parameters. The primary objective of this study was to identify time-resolved changes in bubble dissolution due to softening of a viscoelastic medium. Furthermore, the observed changes in bubble dissolution were correlated with histotripsy treatment outcomes. Additional data on bubble-cloud dynamics were collected in water and compared to the findings in agarose. Water mimics many soft-tissue acoustic properties, including cavitation threshold pressure [24]. Therefore, the study of bubble-cloud dynamics in water has been an important focus in understanding histotripsy-induced cavitation [9]. Models of histotripsy bubble dynamics were also used to gain insights into the observed shift in diffusive behavior throughout exposure, as captured with ultrafast ultrasound imaging.

A secondary goal for this study was to determine differences in the evolution of features tracked with ultrafast imaging throughout histotripsy exposure relative to other quantitative imaging metrics of bubble-cloud dynamics. Passive acoustic imaging indicates the location and strength of acoustic emission sources spatially [25] and is under development to monitor histotripsy [26,27]. The pixel values for passive images are proportional to the intensity of emissions from the bubble cloud and serve as a surrogate for the strain imparted to regions targeted by histotripsy.

## II. METHODS

### A. *In vitro* gel phantom production

Agarose phantoms were produced following an established protocol [28]. Briefly, freshly excised porcine blood was anticoagulated and centrifuged to isolate erythrocytes. Agarose powder (4 g) and sodium chloride (3.6 g) were dissolved in 400 ml of deionized water. The mixture was then heated in a 700-W microwave for 30-s increments until clear. The solution was degassed in a bath sonicator at a temperature of 65 °C for 30 min. The solution was then poured to fill half of an acrylic mold and allowed to solidify. A 5% red blood cell and agarose solution was then pipetted onto the solidified agarose slab in a thin layer (~500 μm thick), after which a final layer of plain agarose was applied and allowed to solidify. Upon solidification, the phantom had an elastic or Young’s modulus of (85.8 ± 11.9) kPa [29], which was similar to that of malignant tissue such as renal cell carcinoma [e.g., (67 ± 138 kPa)] [30].

### B. Experimental setup

**FIG. 1.**
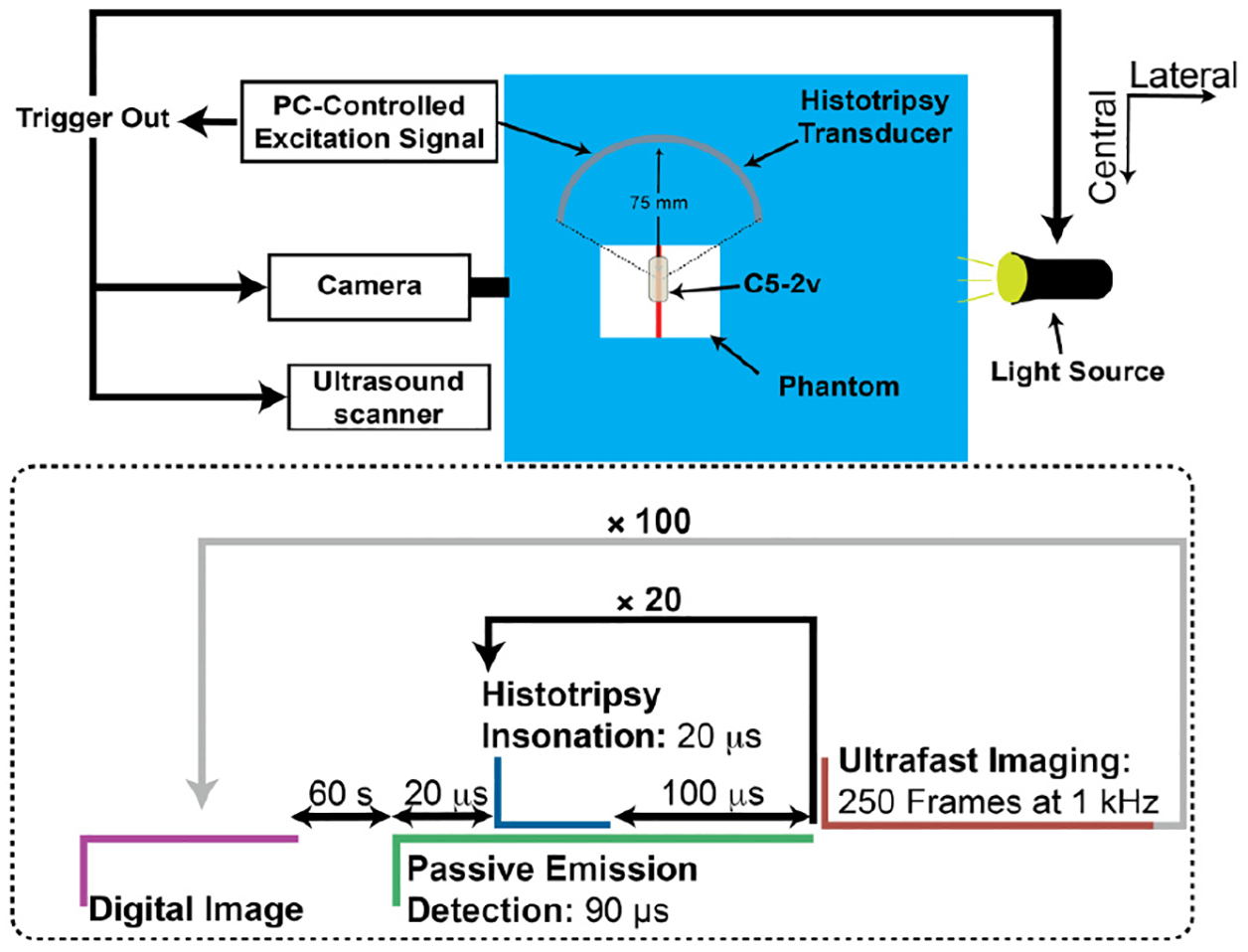
Diagram of experimental setup. Top, setup for data collection. A tissue phantom was exposed to histotripsy pulses with a focused ultrasound transducer. During insonation, a curvilinear imaging probe was used to detect acoustic emissions and track bubble-cloud dissolution with ultrafast imaging. Digital images of the phantom served as the ground truth for locations of histotripsy-induced damage. “Central” (“lateral”) dimensions are relative to the focused transducer. The elevational dimension is perpendicular to the central (lateral) dimensions. Bottom, timing diagram for collection of ultrasound and digital imaging data.

An overview of the experimental setup is shown in Fig. 1. An eight-element focused source was used to generate bubble clouds within the red-blood-cell layer of the agarose phantom. The source had an outer diameter of 10 cm, focal length of 7.5 cm, and fundamental frequency of 1 MHz. Elements in the source were excited with electrical signals generated by a class-D amplifier and matching network. The focal pressure of the source was measured with a fiber-optic hydrophone (HFO-690, Onda Corporation, Sunnyvale, CA) up to pressure levels at the threshold for bubble generation (~25-MPa peak negative pressure). A linear extrapolation between the voltage applied to the amplifier and focal peak negative pressure was used to estimate driving levels beyond the calibration, as described previously [31]. The −6-dB width of the elliptical focal region had dimensions of 5 × 1 × 1 mm^3^ [32].

The phantom was placed in a tank filled with reverse-osmosis filtered (0.2 μm pore size) degassed (~50% *p*O2) water. The focus of the transducer was aligned to a depth of 2 cm in the phantom. Bursts of 20 histotripsy pulses were applied at a rate of 50 Hz to a fixed location within the phantom (Fig. 1). The pulses were 20 μs in duration (20 acoustic cycles) and had an extrapolated peak negative pressure of 35 MPa. This process was repeated 100 times, resulting in a total of 2000 applied pulses. As a control, data were collected in degassed water alone (i.e., without the agarose phantom).

After each burst of 20 histotripsy pulses, a total of 250 ultrafast images were acquired at a 1-kHz rate with a curvilinear imaging probe (C5–2v, Verasonics, Inc, Kirkland, WA) controlled by a research ultrasound scanner (Vantage 128, Verasonics, Inc, Kirkland, WA). The imaging plane of the probe was aligned with the central axis or elevational dimension of the therapy source (Fig. 1). The imaging probe to focal zone distance was 60 mm. To avoid interference between the imaging and therapy pressure pulses, ultrafast imaging data collection was initiated 100 μs after excitation of the histotripsy source.

To increase scatter from the bubble cloud, chirp-coded excitation-based imaging was employed [33]. Rectangularly windowed pulses of 3.7–7.3-MHz bandwidth and 3.6-μs duration were synthesized digitally and applied to each of the 128 elements of the probe in parallel. No apodization was used on the transmit or receive signals. The peak negative pressure of the imaging pulse within the histotripsy focal region was 200 kPa (mechanical index of 0.1 [34]). A matched filter was applied to received signals prior to beamforming, using the transmitted pulse as a template signal. A prior study indicated no difference in the estimated bubble-cloud size when comparing chirp-coded excitation relative to a standard imaging pulse sequence [15].

For passive acoustic imaging, the imaging probe was used to record acoustic emissions generated by the bubble cloud over a 90-μs duration (Fig. 1). The probe started recording signals 20 μs prior to the anticipated focal excitation. Recorded emissions were processed offline with the robust Capon beamformer with a tolerance factor of 10 to assign pixel values [35].

**FIG. 2.**
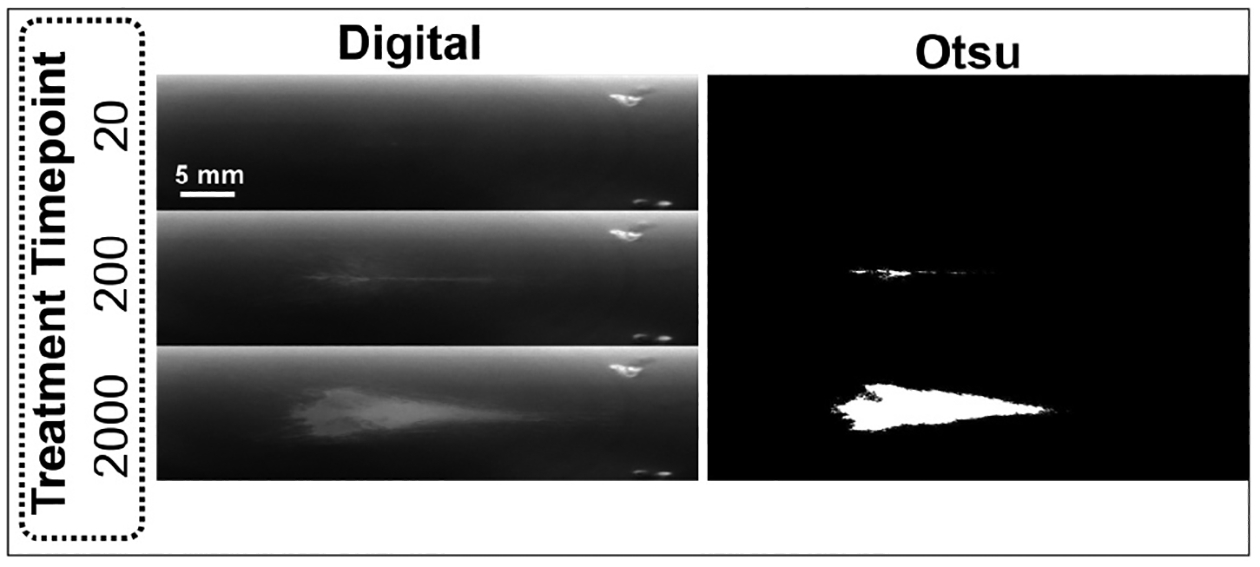
Left, photographs of phantom. Right, segmentation of phantom images. White pixels indicate areas of the ablation zone segmented with Otsu’s method. “Treatment timepoint” indicates the number of applied histotripsy pulses.

After collection of the ultrasound imaging datasets, a 2.8-MP digital CMOS camera (LUCID Vision Labs, Inc., Elf Place, BC) was also triggered to capture a digital image of the red-blood-cell phantom (Fig. 2). The camera was fitted with a lens (50-mm focal length, *f* number 2.5–16, 1stVision Inc, Andover, MA), resulting in an image resolution of about 5 μm per pixel. A pulsed light-emitting-diode array (Luma10, HitLights, Chino, CA) was placed opposite the camera for illumination. Digital images of the phantom were downloaded and processed offline with Otsu’s method to determine the area of damaged phantom (Fig. 2).

### C. Analysis of imaging data

**FIG. 3.**
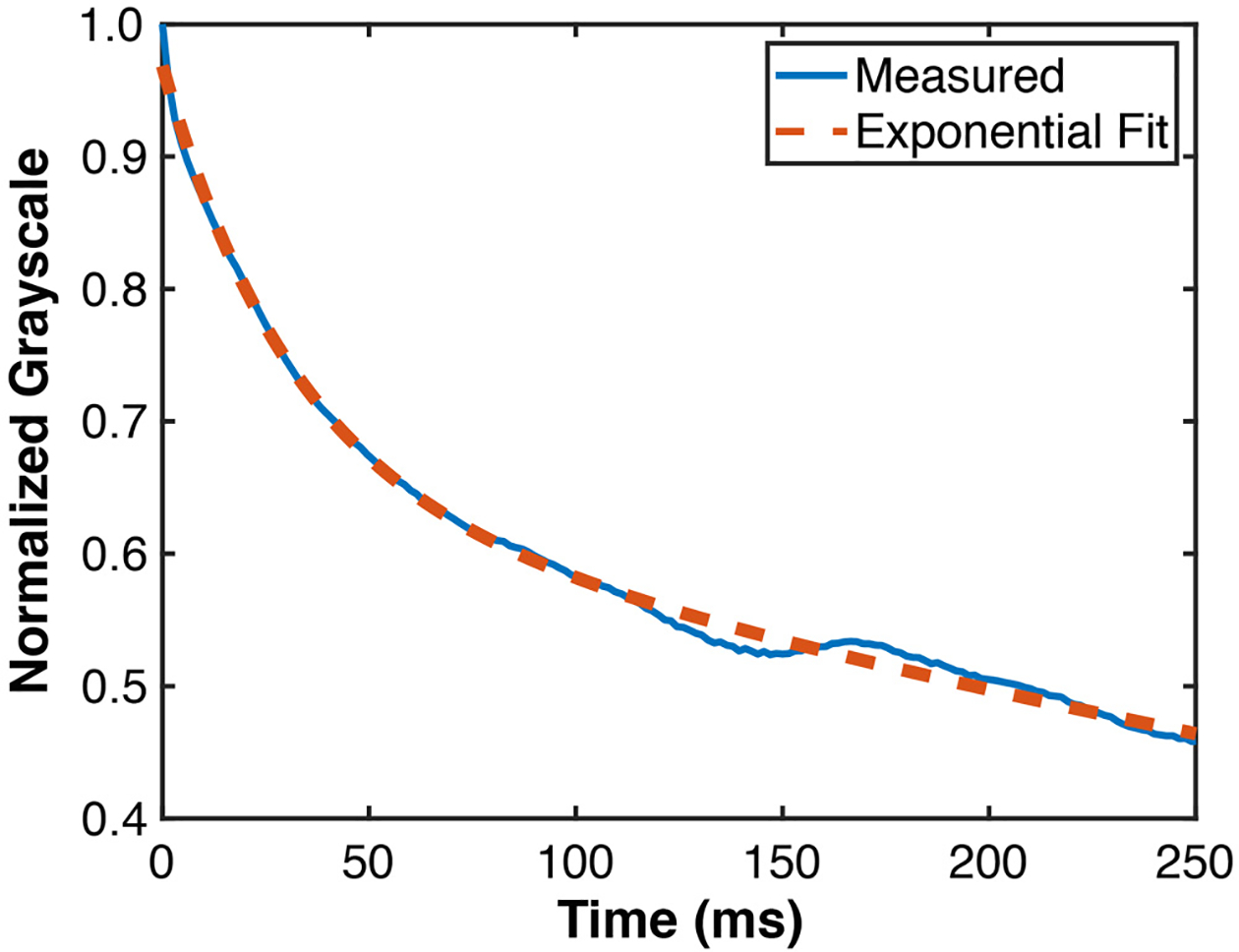
Representative example of the bubble-cloud grayscale intensity over time tracked with ultrafast imaging. Dashed line denotes an exponential fit to data. Here, 1 corresponds to the maximum grayscale intensity (i.e., the maximum scatter of the imaging pulse from the bubble cloud), and 0 indicates no scatter from the bubble cloud (i.e., scatter is equivalent to background without bubble cloud).

For each ultrafast imaging dataset, pixels associated with the bubble cloud were identified with Otsu’s method [36]. The change in average bubble-cloud pixel grayscale intensity over time was fitted in the least-squares sense to an exponential decay of the form

(1) $$\frac{\left\langle I\right\rangle }{\left\langle I_{0}\right\rangle }-{\text{e}}^{-t/a},$$

where *I* is the grayscale intensity of the bubble cloud, *t* is time, and *a* is a fitting parameter with units of time. The subscript “0” indicates the echogenicity at the start of ultrafast-imaging data collection. The angled brackets indicate average values over all bubble-cloud pixels. A representative dataset is indicated in Fig. 3. For each dataset collected throughout histotripsy exposure, the parameter “3*a*” was tabulated as the time to bubble-cloud dissolution (i.e., 〈*I*〉*/*〈*I*_0_〉 ~ 0.05, near the detection limits of the imaging system [33]).

The dissolution time for bubbles in fluids and gels is directly proportional to their radius, *R*_0_ [Eq. (13) in Ref. [37]]:

(2) $$t_{\text{diss}}=\frac{R_{0}^{2}}{L_{8}D}\left(\frac{P_{0}R_{0}}{6\sigma }+\frac{1}{3}\right),$$

where *P*_0_ is atmospheric pressure, *L*_*g*_ is the Ostwald coefficient (1.71 × 10^−2^ [37]), *σ* is the surface tension (0.056 N/m for agarose [38] and 0.072 N/m for water [37]), and *D* is the diffusion constant (1.94 × 10^−9^ m^2^/s for agarose [39] and 2.05 × 10^−9^ m^2^/s for water [37]). The bubble-cloud-dissolution time, *t*_diss_, was set equal to “3*a*” based on Eq. (1). The “root” function in matlab (v2024b, Mathworks, Natick, MA, USA) was used to solve Eq. (2) for *R*_0_. Note that the ultrafast-imaging data were collected after bubbles were forced to expand under the histotripsy pulse, at which point their dynamics are dominated by Fickian diffusion [14]. Therefore, *R*_0_ represents a residual bubble radius characterized as the radius at which the bubble reaches mechanical or diffusive equilibrium.

A cloud of bubbles over a range of sizes are generated by the histotripsy pulse [13]. The calculations performed here used the average bubble-cloud pixel intensity in Eqs. (1) and (2) to estimate the residual bubble size. The predictions here therefore represent the average radius of a bubble within the cloud, and Eq. (2) is used to determine the overall cloud-dissolution time.

### D. Analytic calculations of bubble dynamics

An established analytic model of histotripsy bubble dynamics was used to calculate the maximum bubble radius (*R*_max_) based on the pulsing conditions considered in this study [40]:

(3) $$R_{\text{max}}-\left[R_{n}+\sqrt{\frac{2P_{0}\xi }{9\rho }}\tau \right]{\left[\frac{\xi P_{0}}{3p_{\text{eff}}}+1\right]}^{1/3}.$$

Here, *R*_*n*_ and *ρ* are the bubble-nucleus radius (2.5 nm, as indicated previously [41]) and medium density (1000 kg/m^3^). The terms *ξ*, *τ*, and *p*_eff_ account for viscosity, surface tension, and elasticity based on a Fung-like model, respectively. A previous study demonstrated that Eq. (3) predicted bubble expansion within 14.3% ± 13.0% of experimental measurements conducted in agarose gels performed via the use of high-speed digital photography [40]. An assumption made in the formulation of Eq. (3) was that the histotripsy pulse duration was one cycle, whereas the data collected here used a 20-cycle pulse. Use of Eq. (3) was justified based on the observation that there was little change in the histotripsy bubble dynamics more than one cycle after nucleation [13].

The repeated application of histotripsy will reduce the elastic component of a viscoelastic medium [42]. To gain insights into shifts in the bubble dynamics that resulted from the gradual loss of the elastic component during histotripsy exposure, Eq. (3) was used to calculate the maximum bubble radius from Young’s moduli from 0 to 100 kPa for the histotripsy pulse conditions considered in this study. This range of Young’s moduli spans that anticipated as the phantom undergoes fractionation [e.g., from an initial elastic modulus of (85.8 ± 11.9) to 0 kPa [29]]. The range was extended to 100 kPa for illustrative purposes.

**FIG. 4.**
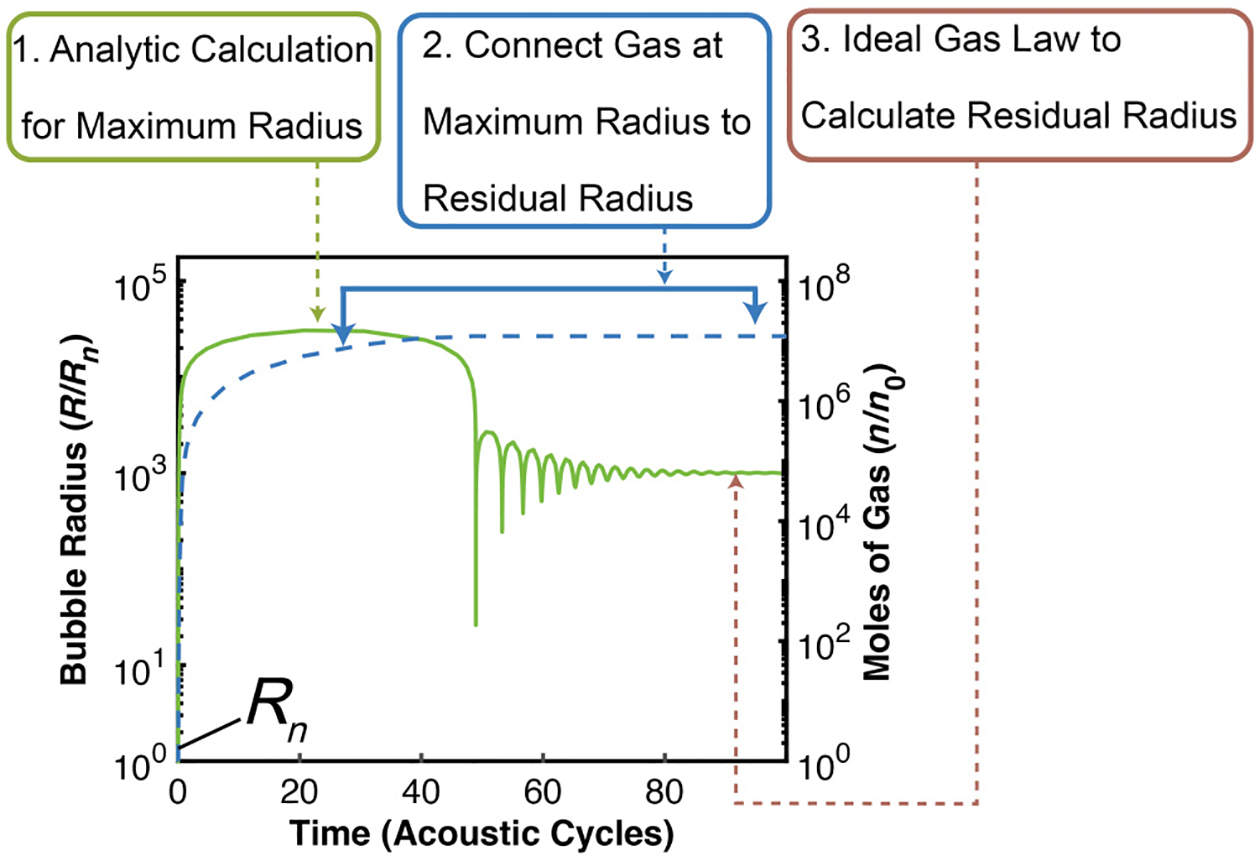
Overview of calculation of the maximum bubble radius following Eq. (3) and the residual radius via solving Eq. (5) for *R*_0_. Presented change in bubble radius and gas content shown were computed numerically with a modified version of the Gilmore equation, as described previously [16]. Bubble-nucleus radius (*R*_*n*_) was 5 nm, consistent with a prior numerical investigation of histotripsy bubbles [41]. Excitation pressure was a 0.75-μs tensile pulse with peak negative pressure of 35 MPa. Note that the bubble-nucleus radius differs from the residual radius due to the extensive influx of gas into the bubble during expansion.

As outlined in Fig. 4, a prior numerical investigation into diffusion during histotripsy identified that the amount of gas in a bubble at its maximum size was reduced by approximately a factor of 2 relative to that after its collapse (i.e., the residual bubble radius denoted as *R*_0_ in Fig. 4) [16]. Note that the residual bubble radius differs from the initial bubble-nucleus radius due to extensive gas influx into the bubble during its expansion phase. A zero-order solution of Fick’s diffusion equation [Eq. (41) in Ref. [43]] was used to determine the number of moles of gas at *R*_max_:

(4) $$n\left(R_{\text{max}}\right)-m_{0}+8C_{0}R_{\text{max}}^{2}{\left(\pi Dt_{\text{max}}\right)}^{1/2},$$

where *C*_0_ is the saturated gas concentration (0.47 mol/m^3^ based on the measured *p*O2 level of the water bath [36]), and *t*_max_ is the temporal duration of bubble expansion (i.e., tensile period of the histotripsy pulse) [41]. While the fundamental frequency of the histotripsy pulse investigated here was 1 MHz, *t*_max_ was assigned a value of 0.75 μs because of the increased tensile-phase duration associated with nonlinear propagation [44]. The term *A*/*B* in Eq. (41) in Ref. [43] can be neglected for histotripsy excitations and result in less than 1% error in the calculation [36]. The term *n*_0_ is the number of moles of gas in the initial bubble nucleus and can be evaluated using the ideal gas law [16]:

(5) $$n\left(R\right)-4\pi \left(P_{0}+\frac{2\sigma }{R}\right)\frac{R^{3}}{3k_{B}T},$$

where *k*_*B*_ is Boltzmann’s constant, and *T* is the temperature (310 K). The variable *R* in Eq. (5) corresponds to the radius of the bubble nucleus, which is thought to be 2.5 nm [24], resulting in *n*_0_ ~ 1.9 × 10^−20^ mol. Note *n*_0_ is roughly 5 orders of magnitude smaller than the other term in Eq. (4). The total gas content at the residual radius can also be computed by evaluating the ideal gas law equation [i.e., Eq. (5)] at *R* = *R*_0_.

The residual radius was determined by equating the left-hand size of Eq. (4) increased by a factor of 2 to Eq. (5) and solving for *R*_0_ with the root function in matlab (v2024b, Mathworks, Natick, MA, USA).

### E. Statistical analysis

The bubble-cloud-dissolution period tracked by ultrafast imaging, the maximum acoustic emission energy assessed by passive acoustic imaging, and the area of damage to the agarose gel were tracked over the course of 2000 applied histotripsy pulses. In total, imaging sets were collected at 100 treatment timepoints in 12 phantoms and 12 water data, resulting in analysis of 2400 total datasets.

For each treatment timepoint, the average and standard deviation were collected for each metric (e.g., bubble-cloud-dissolution period, maximum power of acoustic emissions, and area of phantom damage). Pearson correlation coefficients between each respective imaging metric and the damage area were assessed with the “corr” function in matlab (v2022a, Mathworks, Natick, MA). A Kolmogorov-Smirnov (K-S) test was applied to the treatment-duration dependence for each respective phantom imaging metric and damage area to determine if their function forms were the same using the “kstest” function in matlab. The change in phantom damage area and bubble-imaging metrics as a function of exposure duration were fitted to a piecewise linear function with a custom script in the least-squares sense in matlab [45]. The fit included one inflection point (i.e., two piecewise linear fits were used), which was tabulated for each parameter (e.g., phantom damage area, bubble-dissolution period, or acoustic emissions).

## III. RESULTS

### A. Change in phantom bubble-cloud dynamics over the course of histotripsy exposure

**FIG. 5.**
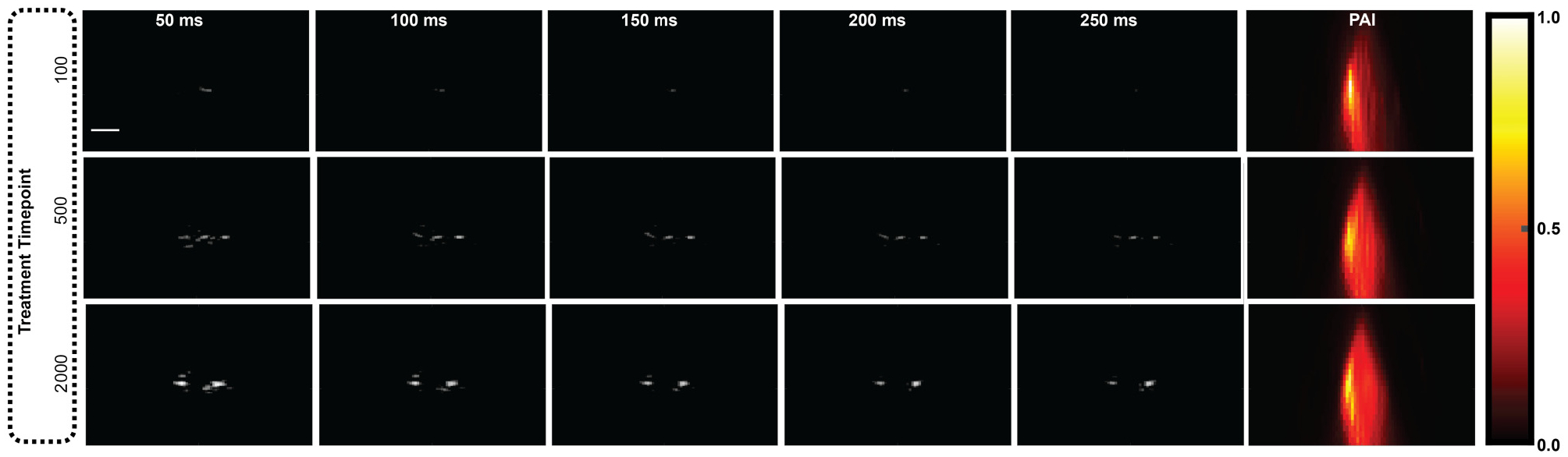
Representative example of the change in bubble dynamics over the course of treatment. Ultrafast images are noted by the grayscale colormap, with the time at which the imaging data were acquired after application of the histotripsy pulse at the top of each column. Passive acoustic images (PAIs) are noted by the last column with pixel values proportional to the acoustic intensity gauged by the color bar. Number of applied pulses is noted by the number on the far left of each row. Over the course of treatment, a larger fraction of the bubble cloud persists for a longer time, relative to baseline, indicating that the bubbles within the cloud are bigger. Note that acoustic emissions are largely unchanged over the explored timepoints. Histotripsy pulse propagated from left to right in these images.

Representative stills collected from ultrafast and passive imaging are shown in Fig. 5. For a given number of applied histotripsy pulses, the bubble-cloud echogenicity reduced throughout the acquisition of ultrafast images. During the initial stages of histotripsy exposure (i.e., less than 100 applied pulses), the bubble cloud could not be discerned from the background after about 200 ms. After more than about 500 applied histotripsy pulses were applied, the bubble-cloud echogenicity was more persistent and pronounced throughout ultrafast image acquisition (Video 1). In contrast, minimal changes were noted in the strength of acoustic emissions tracked by passive acoustic imaging over the course of treatment (Video 2).

**FIG. 6.**
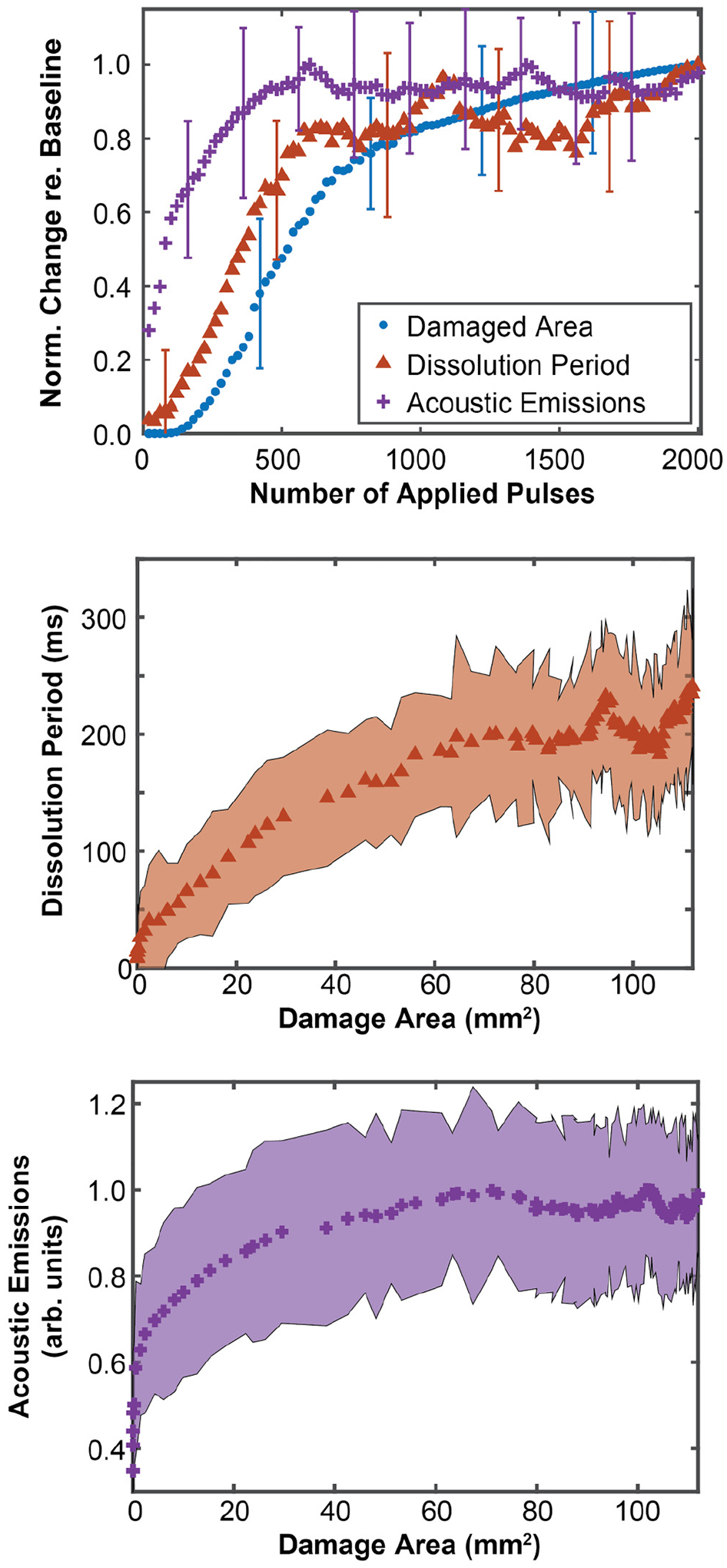
Top, change in phantom damaged areas and bubble-imaging metrics over the course of treatment. All metrics have been compressed to a range of 0 (initial value) to 1 (maximum value). Error bars are representative standard deviations in data (*N* = 12). Middle, correlation between damage area and bubble-cloud dissolution. Bottom, correlation between damage area and the strength of acoustic emissions.

These qualitative observations were consistent with quantitative metrics of bubble clouds, as noted in Fig. 6. Here, the bubble-imaging metrics and damage area in the phantom are reported as percentage increase relative to the baseline and compressed to a dynamic range of 0 to 1 (i.e., *X*_*np*_ = (*X*_*p*_ – *X*_0_)/(*mX* – *X*_0_), where *X*_*np*_ is the normalized compressed metric after *p* histotripsy pulses were applied, *X*_*p*_ is the metric after *p* pulses, *X*_0_ is the bubble-imaging metric for the first timepoint at which data were collected, and *mX* is the maximum value collected for the bubble-imaging metric over the course of treatment).

The changes in phantom damage area, dissolution period, and acoustic emissions over the course of histotripsy exposure were well described by a piecewise linear function. The best-fit value for inflection points and goodness-of-fit metrics are reported in Table I. The inflection point for the phantom damage area was 768 ± 42 applied pulses, suggesting diminishing returns for longer treatment periods. Over the first 555 applied pulses (inflection point for ultrafast imaging), the bubble-dissolution period increased by 40.43% ± 18.8% [(55.8 ± 46.3) ms] relative to the baseline. Over the remainder of the treatment, the bubble-dissolution period increased 53.6% ± 23.1% (80.6 ± 68.7 ms). Acoustic emissions increased 50.5% ± 23.0% over the first 348 applied pulses (inflection point for passive acoustic imaging), and 60.0% ± 20.0% overall.

The Pearson’s correlation coefficients for each imaging metric to the phantom damaged area were found to be significant (*ρ* = 0.94 for dissolution period and damage area, *p* < 0.01; *ρ* = 0.94 for acoustic emission intensity and damage area, *p* < 0.01, Fig. 6). The time dependencies for damage area and dissolution period were found to be equivalent based on a K-S test (*p* > 0.05), but not between the damage area and acoustic emissions (*p* < 0.05).

### B. Bubble-cloud dynamics in agarose and water

**FIG. 7.**
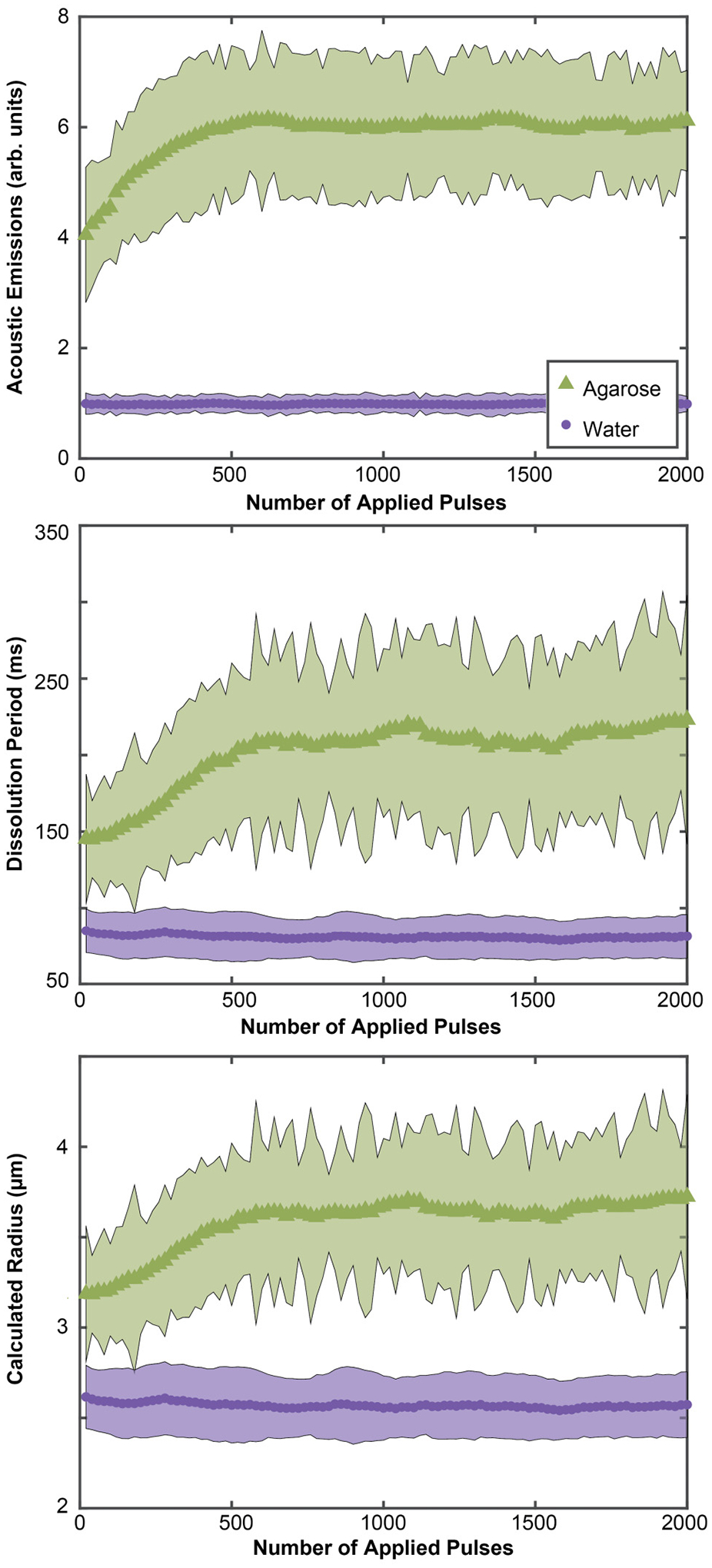
Comparison between bubble-dynamics metrics in a 1% agarose phantom or water. Top, maximum passive acoustic imaging pixel value, which is proportional to the acoustic pressure emissions generated by the bubble cloud; middle, bubble-cloud-dissolution period tracked with ultrafast imaging; and bottom, calculated bubble radius of dissolving bubbles via solving Eq. (2) for *R*_0_. Shading indicative of standard deviations in data (*N* = 12).

Absolute values for acoustic emissions and dissolution period for bubble clouds generated in agarose and water are indicated in Fig. 7. While the bubble-cloud acoustic emissions and dissolution period increased in a piecewise fashion for agarose, there were no noted trends for these parameters over the investigated treatment duration for water datasets (Pearson’s *ρ* = 0.03 and *p* = 0.32 for acoustic emissions; Pearson’s *ρ* = −0.06 and *p* = 0.08 for dissolution period). The bubble-cloud acoustic emissions and dissolution period were increased in agarose relative to water at all points in the histotripsy exposure. There was a strong correlation between the calculated residual bubble radius and dissolution period (Pearson’s *ρ* = 0.986, *p* < 0.01), and the residual bubble radius and acoustic emissions (Pearson’s *ρ* = 0.961, *p* < 0.01) for agarose. There were similar findings for water data: Pearson’s *ρ* = 1.00, *p* < 0.01 between the residual bubble radius and dissolution period, and Pearson’s *ρ* = 1.00, *p* < 0.01 between the residual bubble radius and acoustic emissions.

The residual bubble radius calculated based on the dissolution period and Eq. (2) are also shown in Fig. 7. The residual bubble radius in agarose increased on average by about 14% over the first 555 applied pulses [(3.2 ± 0.4) to (3.5 ± 0.4) μm]. Over the remaining treatment period (555 to 2000 applied histotripsy pulses), the residual bubble radius increased 2.7% to (3.9 ± 0.5) μm. In contrast, there was no noted change in the residual radius of bubbles generated in water over the histotripsy exposure duration [(2.6 ± 0.01) μm].

### C. Bubble-dynamics calculations

**FIG. 8.**
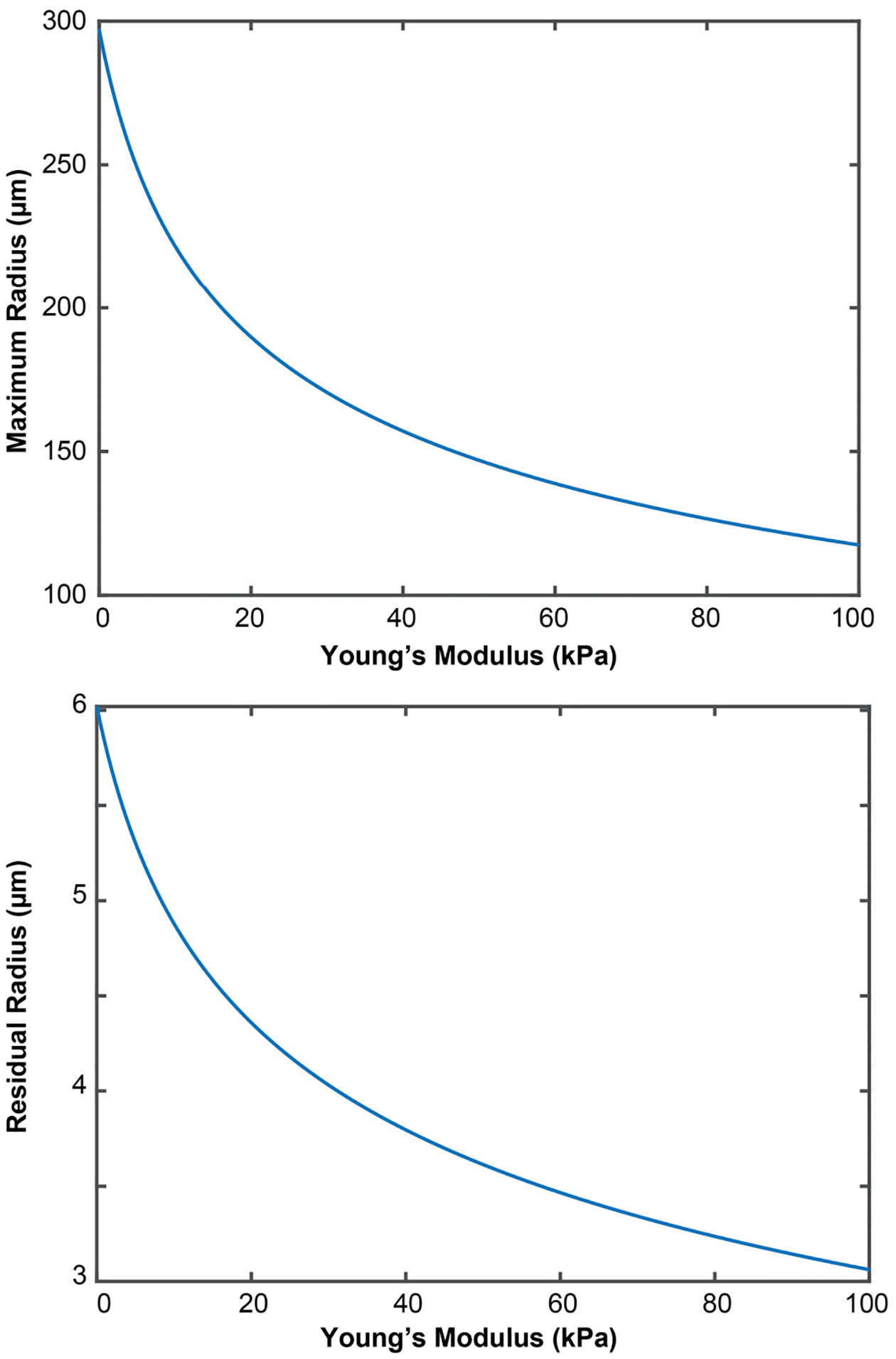
Calculations of (top) maximum bubble radius via Eq. (3) and (bottom) residual bubble radius via solving Eq. (5) for *R*_0_ over an elastic modulus range of 0 to 100 kPa.

The maximum and residual bubble sizes calculated based on Eqs. (3) and (5), respectively, are shown in Fig. 8. Prior measurements of the phantom used in this study indicate the Young’s modulus to be (85.8 ± 11.9) kPa [29,42], which corresponds to calculated maximum and residual bubble radii of 123.8 and 3.2 μm, respectively. Successful histotripsy ablation will result in total liquefaction of the target (i.e., Young’s modulus of 0 kPa) [42]. Comparing the calculations at Young’s moduli of 85.5 and 0 kPa suggest the overall maximum and residual bubble radii should increase to 297.3 μm (140.1%) and 6.0 μm (88.1%), respectively, over the course of histotripsy exposure.

## IV. DISCUSSION

### A. Changes in bubble-cloud-imaging metrics throughout histotripsy exposure

Bubble-induced damage is a consideration for multiple applications [3,46]. The focused ultrasound therapy histotripsy harnesses cavitation to render tissue biologically inert. This study focused on viscoelastic media consistent with the tissue targets of histotripsy [47]. Data were also collected in water, which mimics many acoustic properties of soft tissues and is an important focus when considering histotripsy cavitation [9]. Changes in two primary imaging markers of bubble clouds throughout histotripsy exposure were investigated: dissolution period tracked with ultrafast imaging and acoustic emissions processed with passive acoustic imaging [25,33]. Both reached steady-state values over the 2000 applied histotripsy pulses in agarose. Acoustic emissions approached steady state earlier than the dissolution period (348 and 555 applied histotripsy pulses, respectively). The precise reason for this discrepancy is unknown. Acoustic emissions may be more sensitive than ultrafast imaging to the changes in bubble dynamics during the early stages of treatment. Nevertheless, these data indicate that each imaging metric provides information on different aspects of bubble clouds.

**FIG. 9.**
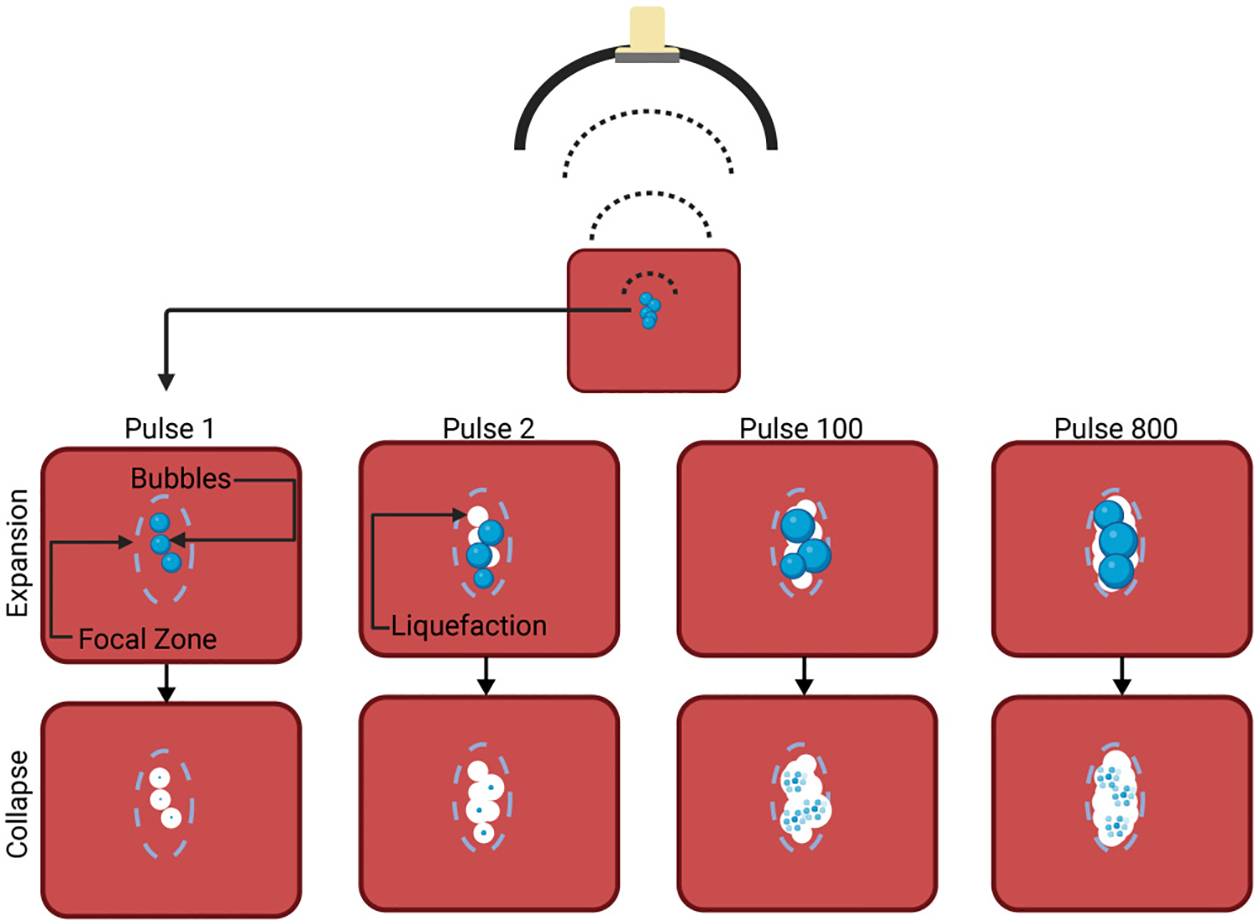
Conceptualization for increased bubble-dissolution period over the course of treatment. Elastic component of the phantom (red square) inhibits bubble expansion. Strains induced by bubbles generated by the histotripsy pulse result in liquefaction of the phantom, resulting in a local reduction in stiffness. During expansion caused by the tension of the histotripsy pulse, there is an influx of gas into the bubble. Larger bubbles entrain more gas, and therefore, take longer to dissolve (pulses 1 and 2). As the bubbles expand further, there may be an instability (e.g., Rayleigh-Taylor) that results in fractionation of the bubbles upon collapse. Pulse number indicated at the top of each column corresponds to both the “expansion” and “collapse” rows.

Ultrafast-imaging data were collected about 100 μs after application of the histotripsy pulse. The observed bubble dynamics were therefore dominated by passive dissolution and not forced by an external pressure pulse [14]. The dissolution period increased with the histotripsy exposure duration (Fig. 6), potentially due to the generation of larger bubbles as the treatment progressed and the medium stiffness decreased [23]. A potential scenario to explain the calculated increase in bubble size with increasing histotripsy exposure is outlined in the “expansion” row of Fig. 9. The precise mechanism of tissue damage under the action of histotripsy-induced bubbles is not fully understood, but it may originate from either compressive strain due to bubble expansion or the strain rate associated with bubble collapse [9]. For either mechanism, bubble expansion is critical. Elasticity acts as an external pressure that reduces bubble expansion [40] and has been shown to reduce the efficacy of histotripsy treatment [48]. Targeted regions soften over the course of treatment [49], thereby leading to greater bubble expansion and therefore a longer dissolution period [23]. A limitation of this study is that the phantom elastic modulus was not measured throughout treatment. However, prior studies have confirmed a reduction in tissue stiffness with histotripsy exposure [42,49].

There were no changes in acoustic emissions or dissolution period in water throughout histotripsy exposure (Fig. 7). In contrast, acoustic emissions and dissolution bubble dynamics were hypothesized to be attributed to a reduction in the phantom elastic modulus throughout histotripsy exposure [42]. The elasticity of a medium suppresses bubble expansion and therefore gas influx (and therefore, the bubble-dissolution period) [23,40] and acoustic emissions [4]. In contrast, water represents a medium where there is no expected shift in elasticity, and therefore, no shift in bubble dynamics.

**FIG. 10.**
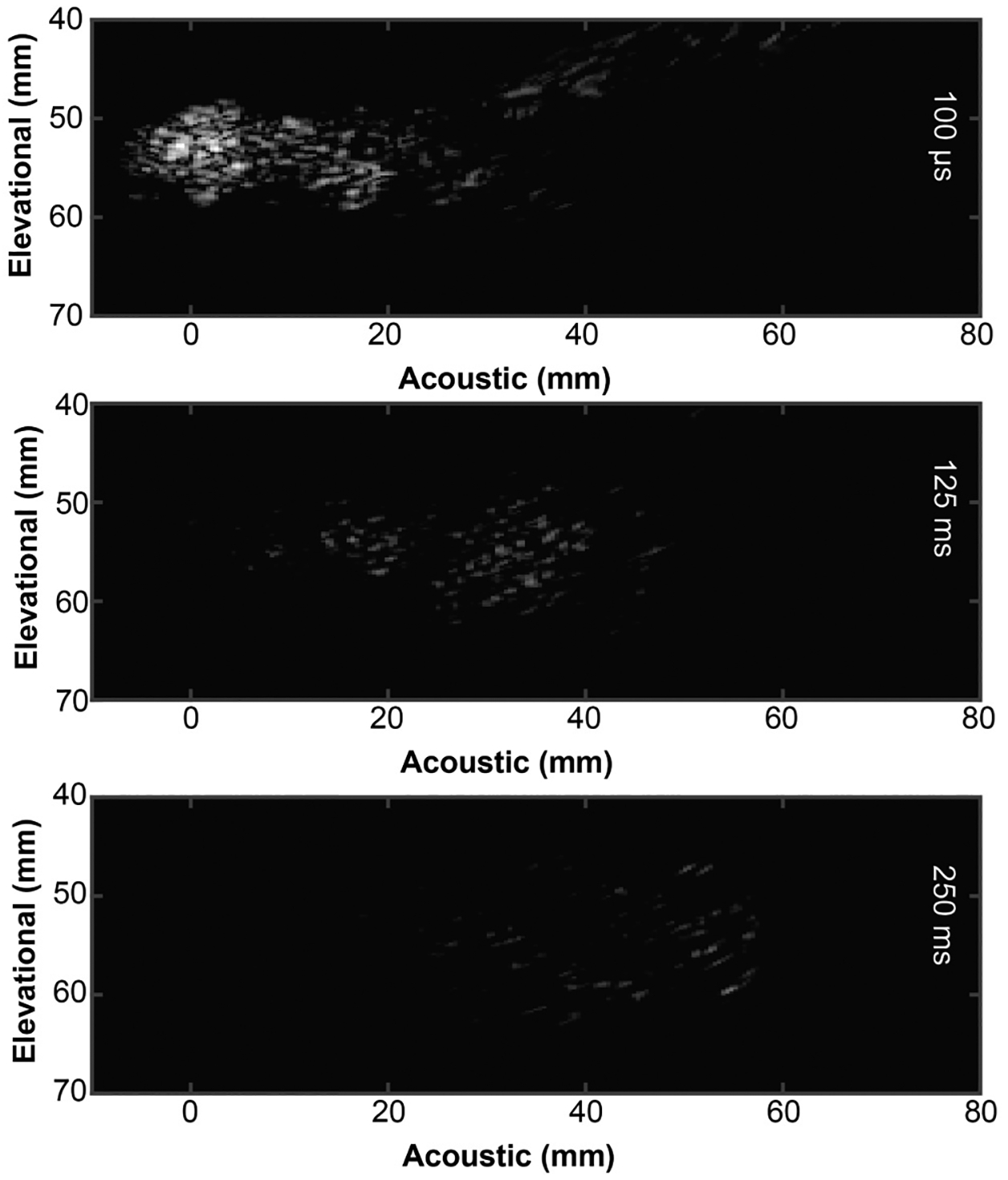
Ultrafast-imaging frames collected of a bubble cloud in water. Focus here is nominally at 0 mm along the acoustic axis and 50 mm along the elevational axis. Note that the bubble shifts nearly 60 mm over the data collection period.

The absolute differences in acoustic emissions and dissolution period for agarose and water warrant consideration. Bubble expansion (and therefore, acoustic emission and dissolution) is anticipated to be maximized in water relative to agarose, although the opposite was observed (Fig. 7). There may be multiple reasons for this discrepancy. Bubble clouds generated in water translated more than a centimeter over the ultrafast ultrasound imaging data acquisition, presumably via acoustic radiation force (Fig. 10) [50]. In contrast, bubbles generated in agarose remained within a millimeter of their initial position throughout the dissolution period (see Fig. 5). The increased translation motion of bubbles within water may result in an increased rate of bubble dissolution. Alternatively, there may be additional forces within the liquefied agarose that mitigate bubble dissolution, which require investigation in future studies.

### B. Correlation of bubble-imaging metrics with outcomes

Thermal forms of ablation rely on the established concept of thermal dose to adjust exposure conditions [51], which can be assessed with magnetic resonance imaging thermometry [52]. Despite moving into routine clinical use [53], histotripsy lacks a bubble-based metric equivalent to thermal dose for the prediction of treatment outcomes. Multiple such metrics are under investigation, including *B*-mode grayscale [26], acoustic emissions [21], and color Doppler [54]. Contrast on *B*-mode imaging and acoustic emissions can be system and sequence dependent [15,33, 55,56]. In this study, the change in emissions over the course of treatment did not have the same functional form as the damaged area (Fig. 6). A modified analysis may provide better methods to use acoustic emissions to gauge treatment outcomes, such as the cumulative power. Color Doppler quantifies fluid motion and has also been shown to change predictably over the course of histotripsy exposure. Doppler measurements of velocity may be confounded in the presence of a dense histotripsy bubble cloud due to local changes in sound speed [57] and artifacts via aliasing or twinkling [58].

Bubble dissolution was investigated in this study as a marker to assess histotripsy ablation. An advantage of the dissolution period over other markers is that it is quantitative, and it has been shown to be independent of processing methods for image formation [15]. A prior study noted the dissolution period may be affected by the imaging pulse pressure [36]. Current guidelines suggest the mechanical index should not exceed 0.4 in the presence of bubbles or other gas bodies [59], which may mitigate these effects. Further studies are needed to determine if other imaging parameters affect the bubble-cloud-dissolution period.

A long-term goal is to develop a real-time imaging metric to gauge the outcomes of histotripsy exposure. The fundamental dynamics of bubble-cloud dissolution based on histotripsy pulsing parameters have been investigated previously [22,36]. This study collected time-resolved data on bubble-cloud dissolution and ablation area throughout histotripsy exposure. The bubble-cloud-dissolution period had the same functional form as histotripsy damage area over the course of treatment (Fig. 6). There was a discrepancy of more than 200 applied histotripsy pulses for the exposure duration at which the bubble-cloud-dissolution period (555 applied pulses) and damage area (770 applied pulses) reached steady-state values (Fig. 6). These data suggest the targeted structure continues to undergo changes, even after the bubble dynamics reach steady-state behavior. It is unclear how to interpret this difference given the *in vitro* nature of this study, making data collection in living tissue a priority for future investigations. Overall, these results support that bubble-cloud-dissolution period provides the feedback necessary that histotripsy exposure duration is sufficient. Specifically, histotripsy exposure can be continued until the onset of a static bubble-cloud-dissolution period (i.e., no change in the dissolution period with the application of additional histotripsy pulses).

Acoustic emissions were not found to have the same function form as histotripsy damage area over the course of treatment (Fig. 6). Prior studies have found that acoustic emissions averaged over the course of histotripsy exposure can be used to predict the extent of histotripsy ablation [18,26,27,29]. The data presented in this study indicate that the change in ablation area lags behind the increase in acoustic emissions. This finding will have an impact on approaches that update the histotripsy exposure scheme based on acoustic emission feedback.

### C. Bubble-dynamics calculations

Total liquefaction of the target occurs when the Young’s modulus of the agarose gel shifts from its initial value of 85.5 kPa to a value of 0 kPa. Calculations based on Eq. (5) suggest the residual radius shifts from 3.2 to 6.0 μm over this range of stiffness (~88% increase). In contrast, estimates based on ultrafast imaging suggest the residual radius increases from (3.3 ± 0.4) to (3.9 ± 0.5) μm, which represents an overall increase of about 16% (Fig. 7). There may be several reasons for this discrepancy. Bubbles may fissure during the later stages of treatment due to increased expansion [60], which is not accounted for in Eqs. (3)–(5). The fissured bubbles would be smaller in size than the initial bubble that underwent collapse and would therefore require a shorter period to dissolve (see the collapse row in Fig. 9). Bubble-bubble interactions, such as coalescence, may alter the dissolution profile and have not been accounted for in this study [61]. Numerical simulations would be required to understand their contributions to the bubble-cloud-dissolution rate [62]. Multiple scattering from the imaging pulse has not been considered, but histotripsy bubble clouds can be very dense and multiple scattering is possible [63]. Furthermore, the bubbly medium will alter the speed of sound and potentially generate artifacts in the imaging data [57]. Future observations with high-speed digital photography are needed to identify the relative contributions of these errors to the overall bubble-cloud-dissolution profile.

### D. Limitations

There are several limitations that prohibit generalization of the findings in this study. A tissue phantom was used to enable a reproducible and consistent medium. Phantoms may not reflect changes in the bubble dynamics that occur in real tissue. The data collected here were for a single phantom formulation. Red blood cells may alter the viscoelastic properties of the phantom relative to agarose alone. A prior analysis of histotripsy bubble dynamics suggested surface tension as the dominant factor that influenced bubble nucleation, and elasticity as a primary contributing factor to overall expansion [9]. The viscosity (5 cP) and surface tension (0.056 N/m) were fixed to values that mimicked the properties of soft tissue for calculations that used Eq. (3). A prior study demonstrated a weak dependence of bubble expansion on viscosity (1–20 cP) and surface tension (0.05–0.08 N/m) [41]. Nevertheless, further consideration is warranted to investigate the influence of histotripsy bubble dynamics based on viscoelastic properties. A Fung-like model was used to model the viscoelastic properties of agarose in Eq. (3). Other models may be more appropriate based on the material, including tissue [64,65]. Additional data in tissue or tissuelike materials with differing elastic properties and stiffnesses will be a focus for future studies. There were no ground truth data to validate the calculations of bubble expansion. However, the analytic model of bubble expansion was previously validated on experimental data [40]. The imaging probe was oriented perpendicular to the therapy source, whereas clinical histotripsy systems align the focused source and imaging probe coaxially [66]. This modified orientation may alter bubble-cloud observations with ultrafast and passive imaging [67]. The effects of temperature change due to ultrasound exposure were not considered in this study. A prior study indicated regions targeted with histotripsy underwent less than 1 °C increase in temperature, which was estimated to alter the phantom diffusivity by less than 1% [68]. Calculations performed in this study focused on models of bubble-cloud dissolution and were most analogous to data collected by ultrafast imaging. There was no model representative of the acoustic emission data assessed by passive acoustic imaging. There is a complicated relationship between bubble size, density, and the resulting acoustic emissions, particularly for the highly nonlinear multicycle shockwave pulses used in this study [69,70]. Future studies may integrate high-level modeling to connect the bubble-cloud behavior to acoustic emissions [71]. The calculations used in this study modeled the behavior of a single bubble, whereas the cloud would be composed of varying bubble sizes [13]. The collected data did not enable an estimation of the bubble-size distribution, but this could be considered in future studies based on attenuation spectroscopy [72]. Despite these limitations, the reported findings indicate the potential of bubble-cloud-dissolution parameters for monitoring histotripsy.

## V. CONCLUSIONS

This study investigated changes in bubble dissolution with repeated formation under the application of focused histotripsy pulses to an agarose gel phantom. Specifically, ultrasound imaging was used to track acoustic emissions and the bubble-cloud-dissolution period. Both imaging metrics reached steady-state values prior to the exposure duration associated with no observed increase in the damaged area. However, the bubble-cloud-dissolution-period dependence on exposure duration had the same functional form as the damaged area. Modeling of bubble dynamics suggested there should be a larger increase in the dissolution period than that observed. This discrepancy was attributed to potential changes in the bubble dynamics not fully captured by the models. Overall, these data indicate that ultrafast ultrasound imaging of bubble clouds provides the information needed to inform an effective histotripsy exposure duration.

## Figures and Tables

**VIDEO 1.** Ultrafast imaging of bubble clouds generated throughout 2000-pulse histotripsy exposure.

**VIDEO 2.** Passive imaging of bubble clouds generated throughout 2000-pulse histotripsy exposure.

**TABLE I.** Summary of piecewise linear fit to changes in datasets with number of applied pulses. Error in these reported metrics represent 95% confidence intervals of the fit for the inflection parameter.

Dataset	Inflection Point (Pulses)	*r* ^2^	RMS Error (%)
Damage area	768 ± 42	0.99	2.8
Dissolution period	555 ± 64	0.96	4.8
Acoustic emissions	348 ± 47	0.95	4.1

## Data Availability

The data that support the findings of this article are not publicly available. The data are available from the author upon reasonable request.
