# Supplementary figures and images for: Changes in bubble-cloud dissolution throughout the application of histotripsy pulses
Source: Phys Rev Appl. Author manuscript; Available in PMC 2026 Jun 27. (PMC13309225; doi:10.1103/9vvg-yfmb)

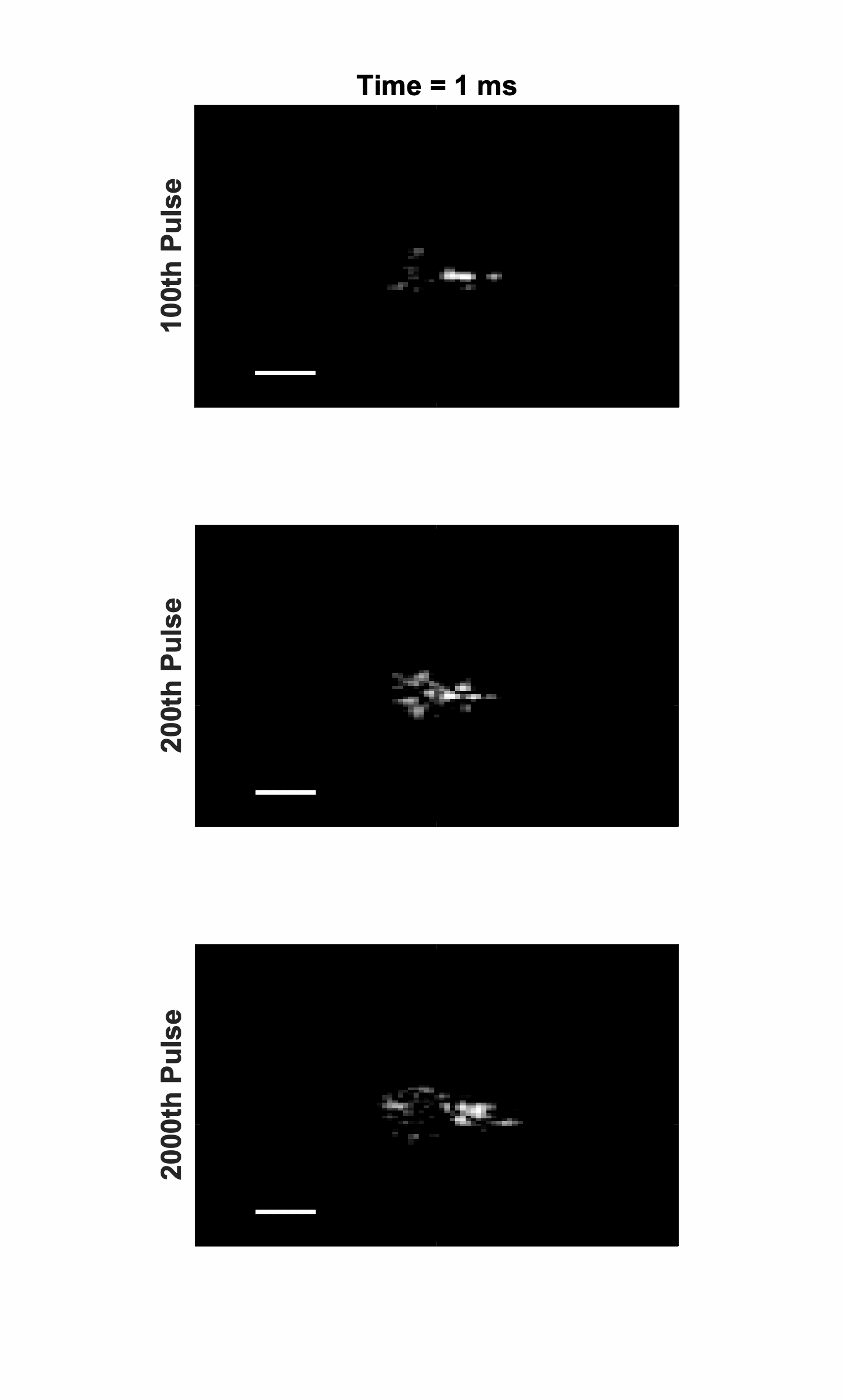

Supplement: Supplementary file 1 [file NIHMS2186650-video-Video_1.gif]
